# Effect of a Supervised Peridialytic Exercise Program on Serum Asymmetric Dimethylarginine in Maintenance Hemodialysis Patients

**DOI:** 10.1155/2020/8878306

**Published:** 2020-10-24

**Authors:** Yaser A. Ammar, Ahmad Awad

**Affiliations:** ^1^Internal Medicine Department, Medical Research Institute, Alexandria University, Alexandria, Egypt; ^2^Chemical Pathology Department, Medical Research Institute, Alexandria University, Alexandria, Egypt

## Abstract

End-stage renal disease (ESRD) patients treated with maintenance haemodialysis (MHD) have alarmingly high atherosclerotic cardiovascular disease morbidity and mortality. Nitric oxide (NO) is the principal endogenous antiatherosclerotic molecule. Increased asymmetric dimethylarginine (ADMA), an endogenous NO synthase inhibitor, was strongly implicated in endothelial dysfunction, premature atherosclerosis, vascular events, and mortality. Regular physical exercise effectively decreased serum ADMA in several patient cohorts, but this potential benefit has not been specifically explored among MHD patients. Forty-four middle-aged ESRD patients treated with thrice-weekly MHD for ≥6 months completed a 6-months regimen of peridialytic lower limb exercise comprising predialytic 10–12 stretching cycles and 20–30 minutes of intradialytic pedaling cycles. Before and after the study, predialytic haemoglobin, serum ADMA, urea, creatinine, calcium, phosphorus, and C-reactive protein (CRP) were measured. Dialysis adequacy was assessed by single-pool Kt/V. The average total physical activity (PA) level was assessed by the International Physical Activity Questionnaire (IPAQ). *P* values <0.05 denoted a statistical significance. The overall level of PA, on both categorical and continuous scales, has significantly increased after application of the exercise program. However, S. ADMA increased from a median of 2375 to 3000 ng/mL (*P*=0.016). Thirty-one patients sustained an increase in S. ADMA (ADMA_Inc), whereas 13 patients had a declining or stable S. ADMA (ADMA_Dec). Compared with ADMA_Inc, ADMA_Dec patients had significantly higher Kt/V (*P*=0.02), higher grade of the basal general PA level (*P*=0.017), and significantly fewer intradialytic hypotension episodes (IDHs) (*P*=0.019). The increase in the S. ADMA and the poststudy S. ADMA level had statistically significant positive correlations with the number of IDHs (*r* = 0.401, *P*=0.007 and *r* = 0.305, *P*=0.044, respectively). A 6-month program of combined aerobic and resistance peridialytic exercise failed to reduce S. ADMA in most MHD patients studied. A modest S. ADMA decline, however, occurred in patients with higher basal PA levels, higher Kt/V, and less IDHs. A potential exercise benefit may be promoted by a multidisciplinary approach targeting increased PA, improved dialysis efficiency, and prevention of IDHs.

## 1. Introduction

Despite advances in haemodialysis (HD) technology, maintenance HD (MHD) patients still suffer an atherosclerotic cardiovascular disease (CVD) risk of 5–10-fold higher than age-matched controls [1]. Nitric oxide (NO) is the principal endogenous antiatherosclerotic molecule, mediating endothelium-dependent vasodilatation and inhibition of platelets adhesion/aggregation and vascular smooth muscle cell proliferation [2]. It is synthesised from L-arginine by the effect of NO synthase (NOS) [3]. Asymmetric dimethylarginine (ADMA) is the principal endogenous competitive inhibitor of the three isoforms of NOS [4]. In experimental models of isolated arterial segments, it inhibited vascular NO production in a concentration-dependent manner [5]. It is synthesised by methylation of arginine residues on histones and other nuclear proteins, catalyzed by protein arginine methyltransferases (PRMTs). Upon proteolysis, ADMA is cleaved from the nuclear proteins and passed to the cytosol and, then, to the plasma [6].

The main pathway (>80%) for ADMA clearance is the enzymatic cleavage by dimethylarginine dimethylaminohydrolase (DDAH), which is abundant in the kidneys, with the remainder being excreted unchanged in urine [7]. Significantly increased levels of circulating ADMA were linked with endothelial dysfunction and demonstrated in patients with different CVD risk factors as hypertension and dyslipidaemia. They were the strongest predictors, beyond traditional risk factors, of cardiovascular events and all-cause and cardiovascular mortality in coronary artery disease patients [8]. Remarkably high levels were described in patients with chronic kidney disease (CKD), starting from its incipient phases [9] and increasing across CKD stages [10] to approach the highest levels ever described among end-stage renal disease (ESRD) patients, particularly those treated with MHD [11]. Increased circulating ADMA emerged as an important link explaining the high CVD morbidity/mortality in CKD patients [12]. Reducing ADMA levels may open a new therapeutic era to reduce CVD burden and complications in these patients [13].

Possibly due to significant protein binding, dialytic clearance of ADMA is much lower than expected from its relatively small molecular weight (202 daltons) [14], and it could not be significantly increased by applying more frequent sessions [15], biocompatible high-flux membranes [16], and either pre- [11] or postdilution haemodiafiltration [17]. No specific pharmacologic approach was successful to upregulate DDAH expression or significantly reduce circulating ADMA by other means [18].

Aerobic exercise training was successful to lower circulating ADMA in some small groups of type 1 [19] and type 2 [20] diabetic patients, coronary artery disease patients [21], and sedentary postmenopausal women [22], though it was not successful in others [23]. Given the extremely low levels of physical activity (PA) in MHD patients compared with age-matched controls [24] and the multiple aspects of health benefits they can achieve by exercise [25], the adoption of an exercise program to lower circulating ADMA in this population is a tempting approach that has not been prospectively studied before.

## 2. Subjects and Methods

### 2.1. Study Design and Participants

**Figure 1:**
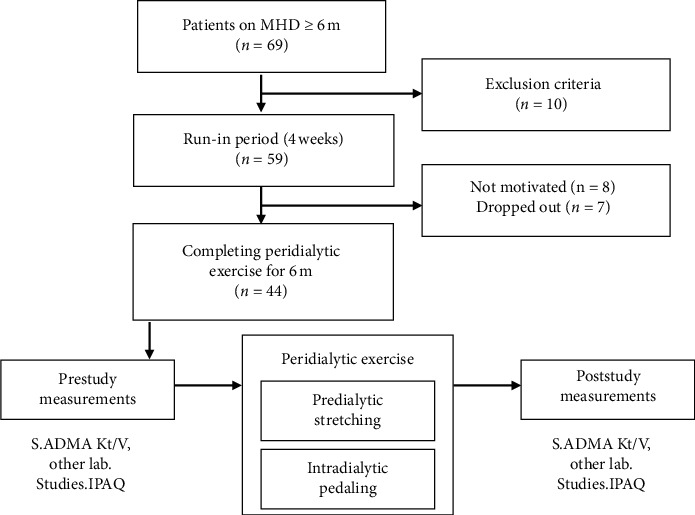
Study flowchart. MHD: maintenance haemodialysis; IPAQ: International Physical Activity Questionnaire.

This was an observational prospective study, conducted in accordance with the Declaration of Helsinki and ethical regulations of the Medical Research Institute, Alexandria University, Egypt. All participants provided written informed consent. Sixty-nine ESRD patients treated with four hourly, thrice weekly, low flux bicarbonate MHD for ≥6 months in the Nephrology Unit, Internal Medicine Department, Medical Research Institute, Alexandria University, Egypt, were evaluated (Figure 1).

Exclusion criteria were as follows:

- Acute coronary syndrome
- Decompensated heart failure
- Unstable haemodynamics (blood pressure <90/60 or >170/110 mm·Hg)
- Advanced cognitive dysfunction
- Advanced mineral bone disease
- Musculoskeletal disorders or disabilities that undermine the patient's ability to perform the exercise

Eligible patients (*n* = 59) were provided a run-in period for 4 weeks to get used for the instruments and build up interest and confidence. Patients not expressing sufficient enthusiasm to accomplish the exercise were excluded. Seven others were dropped out during the study for various reasons and we were left with 44 patients who completed the study (aged 49.5 ± 11 y, 22 males).

### 2.2. Laboratory Studies

The following parameters were assessed in predialytic blood samples obtained twice: at the start of the study and after its completion: haemoglobin level, serum urea, creatinine, calcium, phosphorus, C-reactive protein (CRP), and asymmetric dimethylarginine (ADMA). Postdialytic S. urea was determined to calculate single-pool Kt/V as a measure of dialysis adequacy [26].

The samples for ADMA determination were collected on clot activator serum separator tubes without anticoagulant and, then, centrifuged after ≥10 minutes at 4000 rpm for 15 minutes. The serum was stored at −20°C until analysis which was performed using a competitive ELISA kit [27] (Cloud–Clone Corp., Houston, USA), employing microtiter plates precoated with ADMA. During the reaction, ADMA in the sample competes with a fixed amount of ADMA on the solid phase for sites on the Biotinylated Detection Antibody specific to ADMA. After incubation at 37°C for 45 minutes, excess conjugate and unbound sample or standard were washed. A Horseradish Peroxidase- (HRP-) Streptavidin complex was added to each well and incubated at 37°C for 30 minutes. The microplate was washed again, and a TMB substrate solution was added to each well and incubated at 37°C for 20 minutes. Finally, the enzymatic reaction was terminated by adding sulphuric acid solution, and the optical density was measured spectrophotometrically at 450 nm wavelength. The concentration of ADMA in the samples was, then, determined by comparing the optical density of the samples to the standard curve. The analysis was performed in duplicate. The coefficient of variation was <4%.

### 2.3. Assessment of the Physical Activity (PA) Level

The average ordinary level of PA of all participants was assessed at baseline by filling the formal Arabic language version of the short form of the International Physical Activity Questionnaire (IPAQ), downloadable from http://www.ipaq.ki.se. The questionnaire data were cleaned and truncated as recommended [28]. The results were, then, calculated with the help of an Excel sheet recommended by the IPAQ official site, created by Di Blasio et al. and downloadable at https://www.google.com.eg/url?sa=t&rct=j&q=&esrc=s&source=web&cd=2&cad=rja&uact=8&ved=2ahUKEwi9gJiWnLzkAhX4SBUIHd6YB-kQFjABegQIAxAC&url=https%3A%2F%2Fwww.researchgate.net%2Fprofile%2FPetreanu_Adrian_Ghe%2Fpost%2FCan_anyone_share_the_Hindi_version_of_the_International_physical_activity_questionnaire%2Fattachment%2F59d64ee379197b80779a8253%2FAS%253A494951931039744%25401495017144829%2Fdownload%2FCopy%2Bof%2BIPAQ%2B-%2BAUTOMATIC%2BREPORT%2B-%2BEnglish%2Bversion%2B-%2Bself-admin%2Bshort%2B-%2BDi%2BBlasio%2Bet%2Bal..xls&usg=AOvVaw0na3YCZ9i8-lqUzQfxu0V4.

Two related scores were used to express the total PA:

- Categorical score: PA is expressed as low, moderate, or high.
- Continuous score: the number of metabolic equivalents of the task, or shortly, metabolic equivalents (METs) is a physiological measure expressing the energy cost of PA and is defined as the ratio of the metabolic rate (and, therefore, the rate of energy consumption) during a specific PA to the resting metabolic rate. This rate is multiplied by the activity duration in minutes and, then, the total weekly activities are summed and expressed as (MET. minutes/week) [29].

The activity associated with the peridialytic exercise program (moderate intensity equivalent to 3–5.9 METs/min) was, then, added to the baseline activity level of each patient, and the final (after study) activity level was calculated and expressed on both the categorical and continuous scales.

### 2.4. Peridialytic Exercise [30]

All patients completed a 6-month regimen of supervised graded peridialytic exercise, which was performed thrice weekly inside the dialysis unit and consisted of 2 consecutive phases:

- Predialytic stretching (resistance) exercise: while the patient was sitting in the waiting room before commencement of the dialysis session, he/she was provided with a spring rowing exerciser with 2 handles and 2 foot blades (tummy trimmer) and asked to adapt it between hands and feet and, then, slowly stretch it by bilateral knee extension. The patient is encouraged to do 10–12 cycles of repeated knee flexion and extension at a gradually increasing pace, with short pauses for rest (20–30 seconds) between the cycles.
- Intradialytic pedaling (aerobic) exercise: during the 1^st^ half of the dialysis session, the patient's feet are adapted to a pedal exerciser that allows for adjustment of the resistance level. The patient is asked to do pedaling at a gradually increasing pace for 20–30 minutes.

Verbal encouragement was provided during and after the exercise to increase the patients' motives to approach and maintain a moderate exercise intensity defined as 11–13/20 on Borg's rate of perceived exertion (RPE), described as “somewhat hard” [31] or as “an activity that can be conducted whilst maintaining a conversation uninterrupted” [32]. The exercise was performed under strict observation of the patients' heart rate, blood pressure, and symptoms (if any).

During the study period, any intradialytic hypotensive episodes (IDHs), defined as a fall in the systolic BP of ≥30 mm·Hg to a nadir ≤90 mm·Hg [33], were recorded. Similarly, bacterial infections as defined by typical local, systemic, and laboratory features were recorded.

### 2.5. Statistical Methods

The data were analyzed using SPSS software package version 20 (SPSS Inc., Chicago, Illinois, USA). Continuous variables were tested for normality using the Shapiro–Wilk test. Parametric data were presented as mean ± SD and compared by the paired or independent *t*-test, as appropriate. Nonparametric data were presented as median (interquartile range) and compared by the Wilcoxon signed rank test or Mann–Whitney test, as appropriate. Categorical data were expressed as discrete numbers and compared by the chi-square test or Fisher exact test, as appropriate. Correlations between variables were tested by Spearman's rank correlation coefficient. The significance of results was judged at the 5% level.

## 3. Results

**Figure 2:**
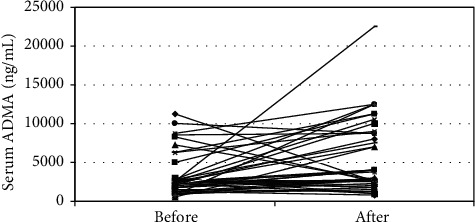
Serum ADMA before and after 6 months of peridialytic exercise.

**Figure 3:**
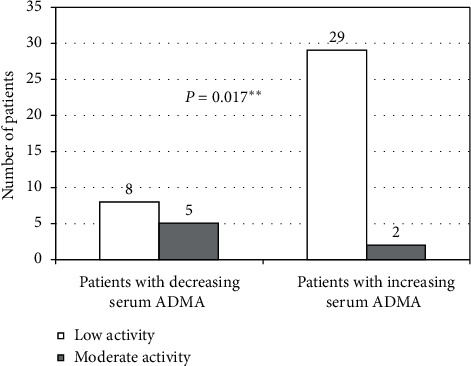
Basal activity level at the start of the study.

**Figure 4:**
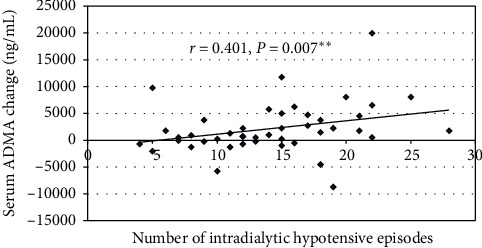
Correlation of the number of intradialytic hypotensive episodes with S. ADMA change.

The overall PA level, on both the categorical and the continuous scales, has significantly increased after application of the exercise program (Table 1). However, S. ADMA has also significantly increased from a median of 2375 to 3000 ng/mL (*P*=0.016). Most other parameters did not have a significant change (apart from an increase of S. phosphorus). Thirty-one patients sustained an increase in S. ADMA (ADMA_Inc), whereas 13 patients had a declining or stable S. ADMA (ADMA_Dec) (Figure 2). Compared with ADMA_Inc, ADMA_Dec patients had significantly higher initial S. ADMA (*P*=0.016), lower final S. ADMA (*P*=0.000), higher Kt/V (*P*=0.02), higher grade of the basal general PA level on the categorical scale (*P*=0.017), and significantly less IDHs (*P*=0.019) (Table 2, Figure 3). The increase in S. ADMA after the study and the poststudy S. ADMA level had statistically significant positive correlations with the number of IDHs (*r* = 0.401, *P*=0.007 and *r* = 0.305, *P*=0.044, respectively) (Table 3, Figure 4). Baseline, S. ADMA had statistically significant positive correlations with S. phosphorus and with Ca × Ph product (Table 3).

An increase in S. ADMA was the prevailing trend, with a median (ICR) change of 1125 (−250 to 3937.5) ng/mL.

Compared with patients with decreasing/stable S. ADMA, those with increasing S. ADMA had a significantly higher proportion of patients with low basal activity level and a significantly lower proportion of patients with moderate activity level.

## 4. Discussion

Patients with advanced CKD, and particularly those on MHD, have greatly reduced exercise capacity and impaired physical functioning [24, 33]. If safely tolerated, they are encouraged to perform PA for, at least, 30 minutes 5 times weekly, as per KDIGO guidelines [34]. However, several medical, social, psychological, and instrumental obstacles limit active exercise participation by MHD patients [35]. The dialysis session itself may provide a good opportunity for a supervised exercise program that invokes better patients' adherence compared with outpatient or home-based programs [36]. The exercise program adopted in this study did result in a significant increase of the PA level of most participants, tripling the ratio of patients with moderate PA from 7 to 21 per 44 (from 16 to 48%, *P* < 0.001) and approximating them to the guideline-recommended PA levels [34]. METs. minute/week also increased significantly (Table 1).

A recent metanalysis of exercise-based interventions in MHD patients revealed that the most common intervention period was 12 weeks (range: 8 to 40 weeks); exercise was mostly performed thrice weekly during the dialysis sessions; the exercise was mostly of moderate intensity and for a session duration ranging from 13 to 90 minutes [37]. Therefore, the cumulative exercise dose instituted in the present study is comparable to most other studies. Heart rate-based adjustment of target exercise intensity level in MHD patients is unreliable and confounded by many factors such as autonomic neuropathy, drug effects (as beta-blockers), and variable fluid status [38]. Alternatively, we monitored exercise intensity by Borg's RPE, which proved to be an affordable and valid tool, independent of patient characteristics, exercise modality, and cardiovascular status [31, 39]. The exercise program was well tolerated and safely completed. A recent meta-analysis of 27 randomized controlled trials of intradialytic exercise concluded that the rate of adverse events was similar in the exercise and control groups [40]. Moreover, the combined (aerobic and resistance) exercise modality we used was reported to be the most effective modality for improving aerobic capacity in MHD patients [41].

The mechanism of exercise-induced ADMA decline is controversial and thought to result from increased ADMA catabolism due to activation of DDAH by a reduction of oxidative stress [42] and a direct stimulation of its gene expression [43]. The failure of the exercise intervention in MHD patients to reproduce the ADMA lowering effect demonstrated in other populations [19–22] has several possible explanations. First, intradialytic exercise has a variable, and occasionally an exacerbating effect, on oxidative stress, which may be attributed to the critically limited antioxidant reserve of MHD patients [44]. Second, exercise intensity/duration might have a putative threshold value below which clinical benefits cannot be achieved [45]. In accord with this, ADMA_Dec patients had comparatively higher baseline (and consequently total) PA level. Third, ADMA accumulation in MHD is quantitatively greater and mechanismally more complex than in other [19–22] patients' groups. In addition to virtual absence of the renal excretory and metabolic ADMA clearance (that cannot be compensated even by the best dialysis techniques available), these patients have markedly increased plasmatic ADMA pooling due to upregulation of PRMT enzymes and increased proteolytic cleavage of ADMA molecules from parent proteins [46]. Resolution of an acute bacterial infection (vascular access-related or otherwise) is characterized by an increased S. ADMA [47]. Such episodes were quite infrequent during the study period (Table 2) and, therefore, cannot be assumed to explain the overall trend of increasing S. ADMA.

The 13 patients with stable/declining S. ADMA (ADMA_Dec) had shown 3 distinguishing features compared with other (ADMA_Inc) patients: higher basal PA level, higher Kt/V, and lower frequency of IDHs. A better dialytic urea clearance reflected by a higher Kt/V is unlikely to be directly reflected on ADMA clearance [15]. Rather, a decline of another uremic toxin involved in ADMA metabolism might be operating. Homocysteine, which can be partially removed by haemodialysis [48], accumulates in MHD patients and increases S. ADMA by increasing proteolysis of ADMA-containing proteins and inhibiting DDAH [49].

An exaggerated S. ADMA elevation in MHD patients prone to IDHs has been reported by several investigators [50, 51]. It has been interpreted as a physiologic response to restore hemodynamic stability [51] and may be mediated, at least in part, by increased protein arginine methylation induced by tissue hypoxia [52]. We also propose that frequent IDHs might be a surrogate marker for dialysis interruption and inefficiency and, thus, retention of homocysteine and other toxins that ultimately increase S. ADMA level.

Each bout of physical exercise creates a circulating “anti-inflammatory” environment [53]. A highly prevalent chronic inflammatory status in MHD is closely linked with malnutrition, atherosclerosis, and mortality [54]. Mitigation of this inflammatory status might be another facet of intradialytic exercise benefits, but longitudinal studies performed in these patients were inconclusive [55], with some reporting a significant reduction in S. CRP in response to exercise intervention [56, 57] and others reporting no change [42, 58]. The 6-month peridialytic exercise intervention of the present study was associated with a decline, albeit insignificant, of S. CRP (Table 1). There was no significant difference in S. CRP between patients with decreasing or increasing S. ADMA (Table 2).

We observed that S. ADMA at baseline had a significant positive correlation with S. phosphorus and Ca × Ph product (Table 3). The significant increase of S. ADMA during the study period was paralleled by a significant increase of S. phosphorus and an insignificant increase of CA × Ph product (Table 1). This came in line with a study of 51 patients with advanced CKD, in whom log plasma ADMA had significant positive correlations with log S. phosphorus, Ca × Ph product, and fibroblast growth factor-23 (FGF-23) [59]. Similarly, within 259 MHD patients, those in higher quartiles of S. ADMA were more likely to have higher S. phosphorus and FGF-23 [60]. Therefore, a relation between increasing circulating ADMA and increasing S. phosphorus and Ca × Ph product in patients with advanced CKD and ESRD likely exists. It remains to be elucidated whether such a relation is mediated through parathyroid hormone [61], FGF-23 [59, 60, 62], or other unidentified factors.

To the best of our knowledge, this study was the first prospective exercise intervention trial targeting to reduce S. ADMA in MHD patients. Although the intervention outcome was not achieved, we proposed several intervening confounders that may be tackled in future studies. The exercise protocol was simple, affordable, well tolerated, and combined both aerobic and resistance exercises. Our study has several limitations. First, we could not include a larger number of participants or a nonintervention control group and could not make other interim measures during the study period. Second, a dedicated physiotherapist was not available to supervise the exercise program. Third, the significantly higher initial S. ADMA in the ADMA_Dec group makes “regression to the mean” a possible concern. However, submitting our work to the flowchart recently suggested by Thomas et al. [63] denoted that this phenomenon “is not obviously a problem.” Indeed, the 2 response groups were equally represented among participants in the upper tertile of initial ADMA values (8 ADMA_Dec and 7 ADMA_Inc; data not shown). Fourth, exercise may improve endothelial function by increasing NO production rather than decreasing S. ADMA [64], a possibility that required assessment of flow-mediated dilatation or NO metabolites. Finally, some variables that may affect the S. ADMA level were not considered as drug treatment, homocysteine level, and residual renal function (though almost all patients were practically anuric).

## 5. Conclusions

A 6-month program of combined aerobic and resistance peridialytic exercise training achieved a significant increase in the general PA level but failed to reduce S. ADMA in most MHD patients studied. A modest S. ADMA decline, however, occurred in patients with higher basal PA level, higher dialysis efficiency (Kt/V), and more stable intradialytic haemodynamics (less frequent IDHs). A potential ADMA-lowering exercise effect may be promoted by a multidisciplinary approach targeting several key elements of MHD patients' care including increased PA, improved dialysis efficiency, and prevention of IDHs. Further studies should apply more intensive exercise protocols on larger numbers of MHD patients and should further explore the multiple confounders that possibly mitigate the exercise benefits. The effect of exercise programs on S. ADMA in patients with less advanced CKD stages should also be explored.

## Figures and Tables

**Table 1:** Comparison of the study parameters before and after the 6-month exercise program

Parameter	Before exercise	After exercise	Statistical test	*P* value
Haemoglobin (gm/dL)	8.82 ± 0.8	8.94 ± 0.7	Paired *t*-test	0.058
S. urea (mg/dL)	102.5 (83–123)	111 (82–136.75)	WSR	0.401
Kt/V	1.03 ± 0.1	1.07 ± 0.1	Paired *t*-test	0.103
S. creatinine (mg/dL)	6.2 (5.2–7.73)	6.8 (5.55–7.93)	WSR	0.183
S. calcium (mg/dL)	8.8 (8.3–9.8)	8.7 (7.68–9.8)	WSR	0.371
S. phosphorus (mg/dL)	5.3 (4.18–5.85)	5.6 (4.98–6.73)	WSR	0.041^*∗*^
Ca × Ph product (mg^2^/dL^2^)	47.17 ± 13.2	51.41 ± 13.9	Paired *t*-test	0.168
S. CRP (mg/L)	8.1 (5.1–13.93)	7.2 (5.1–14.15)	WSR	0.627
S. ADMA (ng/mL)	2375 (1750–2750)	3000 (2500–8188)	WSR	<0.001^*∗∗*^
Physical activity (low/moderate)	37/7	23/21	*χ* ^2^	<0.001^*∗∗*^
Physical activity (METs. minutes/week)	396 (198–792)	576 (378–972)	WSR	<0.001^*∗∗*^

S.: serum, Ca: calcium, Ph: phosphorus, CRP: C-reactive protein, ADMA: asymmetric dimethylarginine, METs: metabolic equivalents, WSR: Wilcoxon signed rank test, *χ*^2^: chi-square test, ^*∗*^significant (*P* < 0.05); ^*∗∗*^highly significant (*P* < 0.01). Continuous data are expressed as mean ± standard deviation (if parametric) or median (interquartile range) (if nonparametric).

**Table 2:** Comparison of patients with decreasing/stable S. ADMA to patients with increasing S. ADMA.

Parameter	Decreasing ADMA (*N* = 13)	Increasing ADMA (*N* = 31)	Statistical test	*P* value
Sex (male/female)	7/6	15/16	*χ* ^2^	0.741
Age (years)	48 (36–54)	52 (43–59.5)	MW	0.321
Duration of dialysis (months)	59 (38–95)	46 (29.5–78.5)	MW	0.589
Haemoglobin (gm/dL)	8.78 ± 0.7	8.83 ± 0.8	Independent *t*-test	0.852
S. urea (mg/dL)	93.46 ± 27.6	108.74 ± 28	Independent *t*-test	0.109
Kt/V	1.09 ± 0.1	1 ± 0.1	Independent *t*-test	0.02^*∗*^
S. creatinine (mg/dL)	5.9 (4.8–7.7)	6.2 (5.3–7.55)	MW	0.487
S. calcium (mg/dL)	8.89 ± 0.9	9.14 ± 1.1	Independent *t*-test	0.452
S. phosphorus (mg/dL)	5.82 ± 1.7	4.97 ± 1.2	Independent *t*-test	0.122
Ca × Ph product (mg^2^/dL^2^)	51.35 ± 15.1	45.42 ± 12.2	Independent *t*-test	0.225
S. CRP (mg/L)	7.7 (5–16.1)	8.1 (5.1–11.75)	MW	0.911
S. ADMA (ng/mL) before exercise	2750 (2500–7250)	2250 (1525–2500)	MW	0.016^*∗*^
S. ADMA (ng/mL) after exercise	2000 (1250–2500)	4000 (2750–9500)	MW	0.000^*∗∗*^
Physical activity before exercise (low/moderate)	8/5	29/2	Fisher exact test	0.017^*∗*^
Physical activity before exercise (METs/week)	495 (396–594)	396 (198–792)	MW	0.517
Physical activity after exercise (low/moderate)	6/7	17/14	*χ* ^2^	0.599
Physical activity after exercise (METs. minutes/week)	675 (576–774)	576 (361.5–972)	MW	0.509
Intradialytic hypotensive episodes	11.31 ± 4.8	15.45 ± 5.6	Independent *t*-test	0.019^*∗*^
Bacterial infections	0 (0–1)	0 (0–1)	MW	0.232

Unless stated otherwise, laboratory parameters are compared for the initial values (before the exercise program). S: serum, Ca: calcium, Ph: phosphorus, CRP: C-reactive protein, ADMA: asymmetric dimethylarginine, METs: metabolic equivalents, MW: Mann-Whitney test, *χ*^2^: chi-square test, ^*∗*^significant (*P* < 0.05). Continuous data are expressed as mean ± standard deviation (if parametric) or median (interquartile range) (if nonparametric).

**Table 3:** Statistical correlations of serum ADMA (before and after exercise) and serum ADMA change.

	S. ADMA before exercise		S. ADMA change		S. ADMA after exercise	
	r	*P*	r	*P*	r	*P*
Age	0.051	0.741	−0.010	0.946	0.06	0.698
Duration of dialysis	0.207	0.177	−0.137	0.374	0.041	0.790
Basal METs. minutes/week (without intradialytic exercise)	0.006	0.967	−0.009	0.954	−0.057	0.711
Intradialytic hypotensive episodes	−0.234	0.127	0.401	0.007^*∗∗*^	0.305	0.044^*∗*^
Bacterial infections	0.278	0.068	−0.172	0.266	−0.069	0.654
Haemoglobin	−0.093	0.547	0.032	0.839		
S. urea	−0.172	0.265	0.047	0.763		
Kt/V	0.249	0.103	−0.261	0.087		
S. creatinine	−0.212	0.166	0.120	0.437		
S. calcium	−0.142	0.357	0.091	0.556		
S. phosphorus	0.372	0.013^*∗*^	−0.081	0.601		
Ca × Ph product	0.330	0.029^*∗*^	−0.024	0.877		
S. CRP	−0.185	0.240	0.149	0.348		

Laboratory parameters studied for correlations are those recorded at the start of the study. S: serum, Ca: calcium, Ph: phosphorus, CRP: C-reactive protein, ADMA: asymmetric dimethylarginine, METs: metabolic equivalents. r: Spearman correlation coefficient, ^*∗*^significant (*P* < 0.05); ^*∗∗*^highly significant (*P* < 0.01).

## Data Availability

The data used to support the findings of this study are available from the corresponding author upon request.

## Supplementary Materials

Supplementary Materials Tables 1 and 2 are represented as supplementary material, with all continuous data expressed as mean ± SD.
