# Supplementary material for: Effect of a Supervised Peridialytic Exercise Program on Serum Asymmetric Dimethylarginine in Maintenance Hemodialysis Patients
Source: Int J Nephrol. 2020 Oct 24;2020:8878306. doi: 10.1155/2020/8878306 (PMC7604598; doi:10.1155/2020/8878306)
Supplement: Supplementary Materials — Tables 1 and 2 are represented as supplementary material, with all continuous data expressed as mean ± SD. [file 8878306.f1.pdf]

Table 1: Comparison of the Study Parameters
before and after the 6-Months Exercise Program

Parameter	Before Exercise	After Exercise	Statistical Test	P Value
Haemoglobin (gm/dL)	8.82 ± 0.8	8.94 ± 0.7	Paired t test	0.058
S. Urea (mg/dL)	104.23 ± 28.5	109 ± 29.5	WSR	0.401
Kt/V	1.03 ± 0.1	1.07 ± 0.1	Paired t test	0.103
S. Creatinine (mg/dL)	6.47 ± 1.6	6.88 ± 1.4	WSR	0.183
S. Calcium (mg/dL)	9.06 ± 1.1	8.8 ± 1.3	WSR	0.371
S. Phosphorus (mg/dL)	5.22 ± 1.4	5.82 ± 1.2	WSR	0.041*
Ca X Ph Product (mg^2/dL^2)	47.17 ± 13.2	51.41 ± 13.9	Paired t test	0.168
S. CRP (mg/L)	13.94 ± 17.7	12.55 ± 13.8	WSR	0.627
S. ADMA (ng/mL)	3205.68 ± 2648.3	5335.23 ± 4502.2	WSR	< 0.001**
Physical Activity (Low / Moderate)	37 / 7	23 / 21	χ^2	< 0.001**
Physical Activity (METs . minutes / Week)	536.25 ± 361.6	708.34 ± 372.8	WSR	< 0.001**

S.: Serum, Ca: Calcium, Ph: phosphorus, CRP: C-reactive protein, ADMA: asymmetric dimethylarginine, METs: metabolic equivalents, WSR: Wilcoxon signed rank test, χ^2: Chi-square test, *: Significant (P<0.05), **: highly significant (P<0.01).
All continuous data are expressed as mean ± standard deviation.

Table 2: Comparison of Patients with Decreasing/Stable S. ADMA to Patients with Increasing S. ADMA

Parameter	Decreasing ADMA (N = 13)	Increasing ADMA (N = 31)	Statistical Test	P Value
Sex (Male / Female)	7 / 6	15 / 16	χ^2	0.741
Age (Years)	46.08 ± 12.8	50.9 ± 10	MW	0.321
Duration of Dialysis (Months)	61.15 ± 32.1	53.74 ± 28.8	MW	0.589
Haemoglobin (gm/dL)	8.78 ± 0.7	8.83 ± 0.8	Independent t test	0.852
S. Urea (mg/dL)	93.46 ± 27.6	108.74 ± 28	Independent t test	0.109
Kt/V	1.09 ± 0.1	1 ± 0.1	Independent t test	0.02*
S. Creatinine (mg/dL)	6.25 ± 1.6	6.55 ± 1.6	MW	0.487
S. Calcium (mg/dL)	8.89 ± 0.9	9.14 ± 1.1	Independent t test	0.452
S. Phosphorus (mg/dL)	5.82 ± 1.7	4.97 ± 1.2	Independent t test	0.122
Ca X Ph Product (mg^2/dL^2)	51.35 ± 15.1	45.42 ± 12.2	Independent t test	0.225
S. CRP (mg/L)	13.35 ± 13.9	14.18 ± 19.2	MW	0.911
S. ADMA (ng/mL) before Exercise	4461.54 ± 3433.7	2679.03 ± 2089.7	MW	0.016*
S. ADMA (ng/mL) after Exercise	2384.62 ± 2030	6572.58 ± 4695.8	MW	0.000**
Physical Activity before Exercise (Low / Moderate)	8 / 5	29 / 2	Fisher Exact Test	0.017*
Physical Activity before Exercise (METs / Week)	594 ± 416.3	512.03 ± 340.6	MW	0.517
Physical Activity after Exercise (Low / Moderate)	6 / 7	17 / 14	χ^2	0.599
Physical Activity after Exercise (METs . minutes / Week)	774 ± 416.3	680.81 ± 356.6	MW	0.509
Intra-Dialytic Hypotensive Episodes	11.31 ± 4.8	15.45 ± 5.6	Independent t test	0.019*

Unless stated otherwise, laboratory parameters are compared for the initial values (before the exercise program). S.: Serum, Ca: Calcium, Ph: phosphorus, CRP: C-reactive protein, ADMA: asymmetric dimethylarginine, METs: metabolic equivalents, MW: Mann Whitney test, χ^2 : Chi-square test, *: Significant (P<0.05). All continuous data are expressed as mean ± standard deviation.
